# National education and implementation initiatives in the field of alcohol and their effect on perceived competence and clinical practice in Swedish primary care

**DOI:** 10.1186/1940-0640-7-S1-A70

**Published:** 2012-10-09

**Authors:** Fredrik Spak, Magnus Geirsson, Marika Holmqvist, Preben Bendtsen, Per Nilsen

**Affiliations:** 1 Department of Social Medicine, Gothenburg University, Gothenburg, Sweden; 2 Sahlgrenska Academy, Unit of Social Medicine, Gothenburg University, Gothenburg, Sweden; 3 Department of Medicine and Health Sciences, Linköping University, Linköping, Sweden

## 

To encourage health professionals to raise the issue of alcohol among patients and to provide better advice to reduce hazardous drinking, Swedish health authorities implemented the Risk Drinking Project (RDP) between 2004-2009. The main activities were training seminars focusing on teaching motivational interviewing and screening for risky drinking. To evaluate the impact of RDP, baseline and follow-up surveys (in 2006 and 2009) were conducted. Participants were general practitioners (GPs) and district nurses (DNs). They were asked how often they discussed alcohol with their patients, and how they estimated their skill at providing advice about drinking and their effectiveness in helping patients reduce risky drinking. We triangulated the results with two population surveys where patients reported whether they had been asked about alcohol when visiting their primary health care (PHC) provider. We also studied changes in the number of alcohol-related diagnoses in PHC in western Sweden between 2005- 2009. Fifty-five percent of PHC providers in the 2009 follow-up survey reported participating in alcohol-related education in the past three years. For all three parameters analyzed (frequency, skill, and effectiveness), there were significant increases during the three years, particularly among DNs. However, the population surveys showed no change in patients being asked about their alcohol consumption. Further, there was only a small increase in alcohol-related diagnoses over this time-period (9%). The RDP is a likely cause of enhanced self-perceived competence among nurses and GPs. Using a combination of data sources to evaluate the impact of RDP raises uncertainty as to whether the educational effort alone was sufficient to increase screening and brief intervention.

